# Non-Additive Optical Response in Transition Metal Dichalcogenides Heterostructures

**DOI:** 10.3390/nano12244436

**Published:** 2022-12-13

**Authors:** Marwa A. El-Sayed, Andrey P. Tselin, Georgy A. Ermolaev, Mikhail K. Tatmyshevskiy, Aleksandr S. Slavich, Dmitry I. Yakubovsky, Sergey M. Novikov, Andrey A. Vyshnevyy, Aleksey V. Arsenin, Valentyn S. Volkov

**Affiliations:** 1 Center for Photonics and 2D Materials, Moscow Institute of Physics and Technology, 9 Institutsky Lane, Dolgoprudny 141700, Russia; 2 Department of Physics, Faculty of Science, Menoufia University, Shebin El-Koom 32511, Egypt; 3 Photonics and Quantum Materials Department, Skolkovo Institute of Science and Technology, 3 Nobel, Moscow 143026, Russia

**Keywords:** transition-metal dichalcogenides, two-dimensional materials, optical constants, heterostructure, refractive index, nanophotonics, spectroscopic ellipsometry

## Abstract

Van der Waals (vdW) heterostructures pave the way to achieve the desired material properties for a variety of applications. In this way, new scientific and industrial challenges and fundamental questions arise. One of them is whether vdW materials preserve their original optical response when assembled in a heterostructure. Here, we resolve this issue for four exemplary monolayer heterostructures: MoS_2_/Gr, MoS_2_/hBN, WS_2_/Gr, and WS_2_/hBN. Through joint Raman, ellipsometry, and reflectance spectroscopies, we discovered that heterostructures alter MoS_2_ and WS_2_ optical constants. Furthermore, despite the similarity of MoS_2_ and WS_2_ monolayers, their behavior in heterostructures is markedly different. While MoS_2_ has large changes, particularly above 3 eV, WS_2_ experiences modest changes in optical constants. We also detected a transformation from dark into bright exciton for MoS_2_/Gr heterostructure. In summary, our findings provide clear evidence that the optical response of heterostructures is not the sum of optical properties of its constituents.

## 1. Introduction

**Figure 1:**
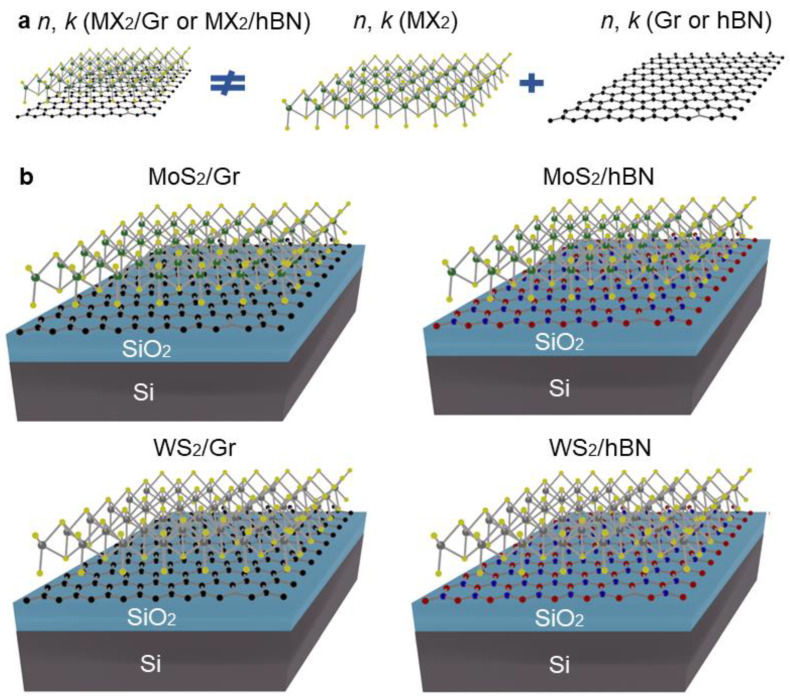
(**a**) Schematic illustration of non-additive optical response of heterostructures. (**b**) Heterostructures used in the work: MoS_2_/Gr, MoS_2_/hBN, WS_2_/Gr, and WS_2_/hBN. All vdW materials are single layers. Green, grey, yellow, black, red, and blue circles label Mo, W, S, C, B, and N atoms, respectively.

The foundation of contemporary solid state physics and the industry is semiconductor heterostructures [1,2]. They offer a unique opportunity to manipulate fundamental material parameters such as bandgap width [3,4], charge carrier mobilities [5,6], and refractive index [7,8]. However, the majority of heterostructures are produced by expensive and complex epitaxial growth [9,10], which is also limited to mixing crystals with less than 1% lattice misfit [11]. On the other hand, dangling bond-free surfaces and van der Waals (vdW) forces enable unrestricted stacking of two-dimensional (2D) materials [12,13,14], which can be controlled via microspectroscopy [15,16]. In electrical and optoelectronic applications, vdW heterostructures have already demonstrated their great value [17,18,19,20]. For instance, graphene (Gr) on top of hexagonal boron nitride (hBN) demonstrates record-breaking carrier mobility of over 10^5^ cm^2^V^−1^s^−1^ [21,22]. Additionally, 2D materials can be placed at various orientation angles, providing an indispensable degree of freedom to further adjust the properties of vdW materials [23,24,25,26]. Among the greatest examples are band structure modification [27,28], interlayer excitons [29,30], and unconventional superconductivity in magic-angle graphene superlattices [31,32]. The use of vdW heterostructures for optical engineering has also been suggested by recent theoretical research [33,34,35,36]. Several studies have also reported that when Gr is included in heterostructures, its intrinsic absorption can grow by up to 60% [37,38]. These results raise the fundamental question of non-additive optical effects in heterostructures (Figure 1a). In other words, we can utilize pristine optical constants of 2D materials [39,40] in vdW heterostructures.

In this study, we investigated this issue for four exemplary heterostructures, depicted schematically in Figure 1b: MoS_2_/Gr, MoS_2_/hBN, WS_2_/Gr, and WS_2_/hBN. We discovered that non-additive effects arise in vdW heterostructures. Still, the change in optical constants strongly depends on the constituents of the heterostructures.

## 2. Materials and Methods

### 2.1. Materials

Full area coverage two-dimensional heterostructures of MoS_2_/Gr, MoS_2_/hBN, WS_2_/Gr, and WS_2_/hBN were purchased from SixCarbon (6Carbon Technology, Shenzhen, China), where monolayers of MoS_2_, WS_2_, graphene and hBN were grown via chemical vapor deposition (CVD) method [41], and subsequently, monolayers of each material were transferred using the standard water-assisted method [42] to form the desired heterostructures on a Si wafer substrate with silicon dioxide.

### 2.2. Raman Characterization

Horiba LabRAM HR Evolution confocal scanning Raman microscope (Horiba Ltd., Kyoto, Japan) was employed for acquisition of the Raman spectra of heterostructures. All measurements were carried out under linearly polarized excitation at free space wavelengths of 532 nm with a 1800 lines/mm diffraction grating and a ×100 objective with 0.90 numerical aperture. The spot diameter was approximately 0.9 µm. The Raman spectra were recorded at an incident laser power of 1.75 mW and an integration time of 10 s. At least 10 spectra were collected from each sample.

### 2.3. Spectroscopic Ellipsometry Characterization

Spectroscopic ellipsometer (VASE, J.A. Woollam Co., Lincoln, NE, USA) was used to determine optical constants of transition metal dichalcogenides (MoS_2_ and WS_2_) in heterostructures. For ellipsometry spectra analysis, we used WVASE software and described the heterostructure with a four-layer optical model (from bottom to top): Si substrate, SiO_2_, Gr or hBN, and MoS_2_ or WS_2_. Optical constants for Si, SiO_2_, Gr, and hBN were taken from previous research [43,44,45]. The thicknesses of SiO_2_ layers are 11.9, 11.8, 317.9, and 328.0 nm for MoS_2_/Gr, MoS_2_/hBN, WS_2_/Gr, and WS_2_/hBN heterostructures, respectively. At the same time, optical response of MoS_2_ and WS_2_ was modeled as a sum of Tauc-Lorentz (TL) oscillators with varied parameters. The formula for the imaginary part of the TL oscillator dielectric permittivity $\varepsilon_{2}$ reads as [46,47]:

(1) $$\varepsilon_{2}=\{\begin{array}{c} \frac{1}{E}\cdot \frac{AE_{0}C{\left(E-E_{g}\right)}^{2}}{{\left(E^{2}-E_{0}^{2}\right)}^{2}+C^{2}E^{2}}\;\mathrm{for}\;E>E_{g}\\ 0\;\;\;\;\;\;\;\;\;\;\;\;\;\;\;\;\;\;\;\;\;\;\;\;\;\;\;\;\;\mathrm{for}\;E<E_{g} \end{array}$$

where E is the photon energy, A is the oscillator strength, C is the oscillator broadening, $E_{g}$ is the optical bandgap, and $E_{0}$ is the oscillator central energy, while the real part $\varepsilon_{1}$ of the dielectric function is derived from Kramers–Kronig integration plus $\varepsilon_{\infty }$ to account for high energy electronic transitions.

### 2.4. Reflectance Measurements

The spectroscopic reflection measurements were performed in the 1.65–2.48 eV (500–750 nm) spectral range on a reflectometer FTPadv integrated into the spectroscopic ellipsometer Sentech SE 800E (SENTECH Instruments GmbH, Berlin, Germany). The reflected light was collected in a backscattering configuration using a focused objective. We measured the reference and background radiation using the supplied reference sample (silicon wafer with 3 nm silicon dioxide). Then, the reflection data were collected from the sample.

## 3. Results and Discussion

### 3.1. Non-Additive Optical Effects in Phonon Spectra

In contrast to previous research [48,49,50] on exfoliated samples with small lateral dimensions of about 10 μm, we focused on large-scale vdW heterostructures since they are more likely to satisfy the demands of practical applications. With this intention, our samples were synthesized via chemical vapor deposition (CVD) [41] and then wet-transferred [42] on each other to form the desired heterostructures (Figure 1) with lateral sizes of around 1 × 1 cm^2^. Furthermore, CVD-grown crystals have random orientations of crystallographic axes [51], which lead to a random distribution of orientation angles between top and bottom monolayers. Hence, the interaction between layers in our vdW heterostructures is averaged over the orientation angle. To investigate the impact of interlayer interaction on optical properties of 2D materials, we performed spectroscopic ellipsometry [40,52,53] and Raman spectroscopy [54,55] measurements.

**Figure 2:**
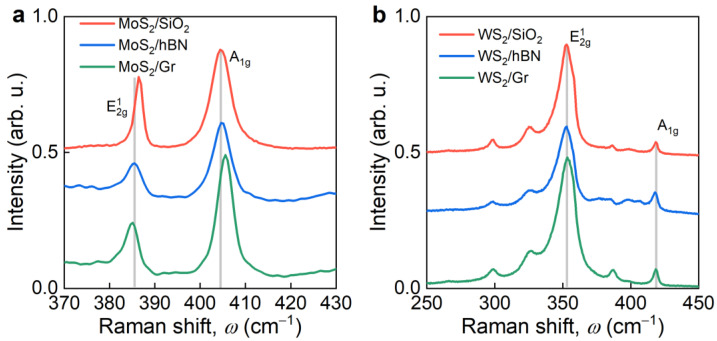
Raman spectra of (**a**) MoS_2_ and (**b**) WS_2_ in heterostructures. Grey lines are guidelines for eyes.

According to Raman measurements (Figure 2a), MoS_2_/Gr and MoS_2_/hBN heterostructures exhibit a noticeable difference in MoS_2_ spectra. For MoS_2_/Gr heterostructure, the E^1^_2g_ mode has a tiny blue shift by 0.5 cm^−1^, whereas the A_1g_ mode red shifted by 1 cm^−1^, in comparison with MoS_2_/hBN heterostructure (Figure 2a). We also included in Figure 1a the Raman spectrum of the MoS_2_ monolayer on a standard silicon dioxide (SiO_2_) substrate as a reference. Predictably, this spectrum is closer to MoS_2_/hBN than to MoS_2_/Gr because SiO_2_ and hBN are both dielectrics, while Gr is semimetal. Based on the difference between the positions of the Raman peaks, we concluded that MoS_2_ optical constants should have some change in the MoS_2_/hBN heterostructure, compared to the classical MoS_2_/SiO_2_, and even greater modification is anticipated for the MoS_2_/Gr heterostructure. Conversely, Raman spectra of WS_2_ do not change in all three cases (Figure 1b). Therefore, we expected minimal changes in WS_2_ optical constants in the WS_2_/Gr and WS_2_/hBN heterostructures.

### 3.2. Non-Additive Optical Effects in Optical Constants

**Figure 3:**
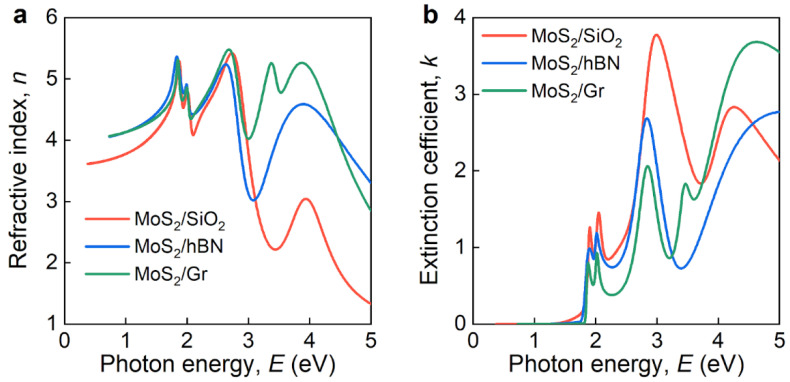
Optical constants of monolayer MoS_2_ in heterostructures. (**a**) Refractive index and (**b**) extinction coefficient.

**Figure A1:**
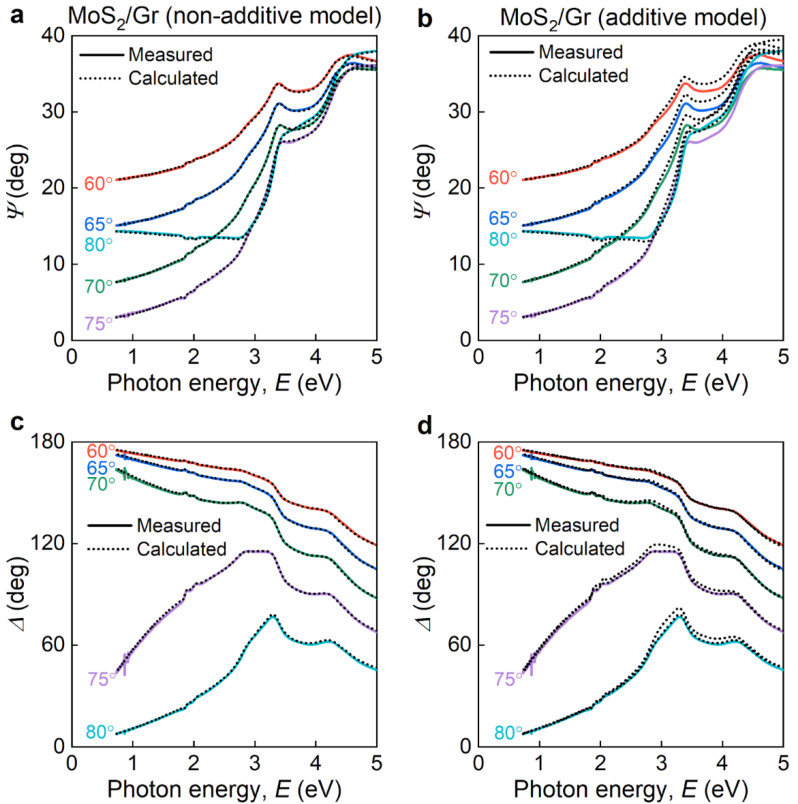
Ellipsometry spectra of MoS_2_/Gr heterostructures. Comparison of non-additive and additive optical models for (**a**,**b**) *Ψ* and (**c**,**d**) Δ. Red, blue, green, violet, and cyan lines correspond to 60°, 65°, 70°, 75°, and 80° incident angles, respectively.

**Figure A2:**
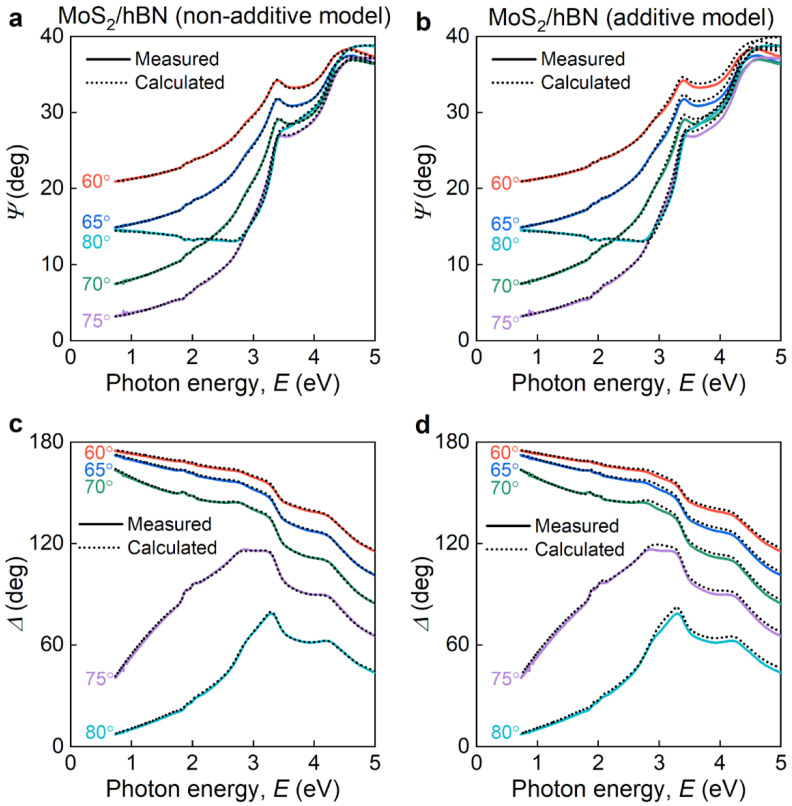
Ellipsometry spectra of MoS_2_/hBN heterostructures. Comparison of non-additive and additive optical models for (**a**,**b**) *Ψ* and (**c**,**d**) Δ. Red, blue, green, violet, and cyan lines correspond to 60°, 65°, 70°, 75°, and 80° incident angles, respectively.

The dielectric response of 2D MoS_2_ in heterostructures was investigated using spectroscopic ellipsometry. A four-layer model (air/MoS_2_/Gr/SiO_2_/Si and air/MoS_2_/hBN/SiO_2_/Si) was employed to retrieve an optical response of the MoS_2_ monolayer in heterostructures from ellipsometry spectra (Figure A1 and Figure A2). In the first step, we performed preliminary ellipsometry analysis, in particular, estimation of silicon oxide thickness. For this purpose, we fitted SiO_2_ thickness within the additive optical model for heterostructures. It gave 11.9, 11.8, 317.9, and 328.0 nm SiO_2_ thicknesses for the MoS_2_/Gr, MoS_2_/hBN, WS_2_/Gr, and WS_2_/hBN heterostructures, respectively. Afterward, we utilized point-by-point fitting [56,57] and then proceeded with the Tauc–Lorentz (TL) oscillator model to adequately describe the MoS_2_ excitons (see Section 2) [46,47]. In accordance with the Raman results, optical constants of MoS_2_ show a dramatic change in heterostructures (Figure 3). First, fundamental A- and B-excitons have lower oscillator strength in MoS_2_/hBN and MoS_2_/Gr heterostructures relative to pristine MoS_2_, although peak positions remain the same. These observations can be explained by the change of dielectric environment, which is known to strongly modify transition metal dichalcogenides (TMDCs) excitons [58,59,60]. Moreover, a recent study [61] established a charge transfer in MoS_2_/Gr, which can explain changes for A- and B-excitons since it could be considered as doping, which is known to modify A- and B-excitons [57]. For high-order MoS_2_ excitons, the situation is more intriguing since they alter not only oscillator strength in MoS_2_/hBN and MoS_2_/Gr heterostructures, but also their position, and doping does not affect them [57]. These changes manifest in substantial difference in the refractive index (Figure 3a) and extinction coefficient (Figure 3b) of MoS_2_ above 3 eV. Moreover, in the MoS_2_/Gr case, we detected an additional excitonic peak around 3.5 eV (Figure 3b). This exciton has previously been observed for MoS_2_ in the presence of organic molecules, turning dark (light-inaccessible) excitons into bright ones (light-accessible) [47,62]. By analogy, we assumed that it is similar for our scenario, except that, instead of organic molecules, it is graphene that assists in turning a dark exciton into a bright one. Additionally, Figure A1 and Figure A2 compare of the non-additive (modified optical response of MoS_2_ by heterostructure) and additive (pristine optical response of MoS_2_) optical models, which show that both methods can be implemented up to 3 eV. Nonetheless, the non-additive model far more accurately captures the experimental results above 3 eV. Therefore, we drew the conclusion that MoS_2_/Gr and MoS_2_/hBN heterostructures can be viewed as independent layers for low-energy photons (<3 eV), whereas vdW interaction needs to be taken into consideration for high-energy photons (>3 eV).

**Figure 4:**
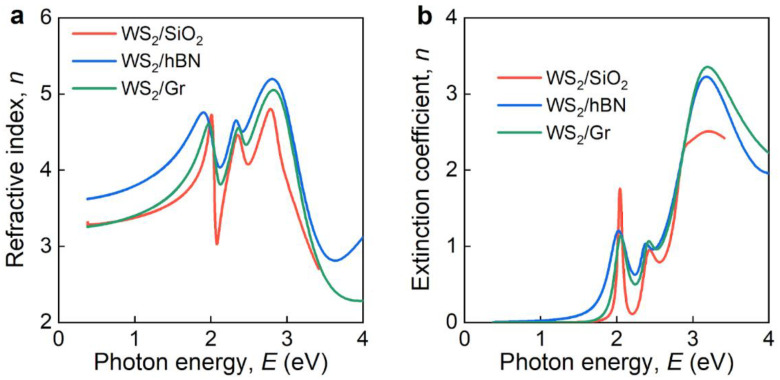
Optical constants of monolayer WS_2_ in heterostructures. (**a**) Refractive index and (**b**) extinction coefficient.

**Figure A3:**
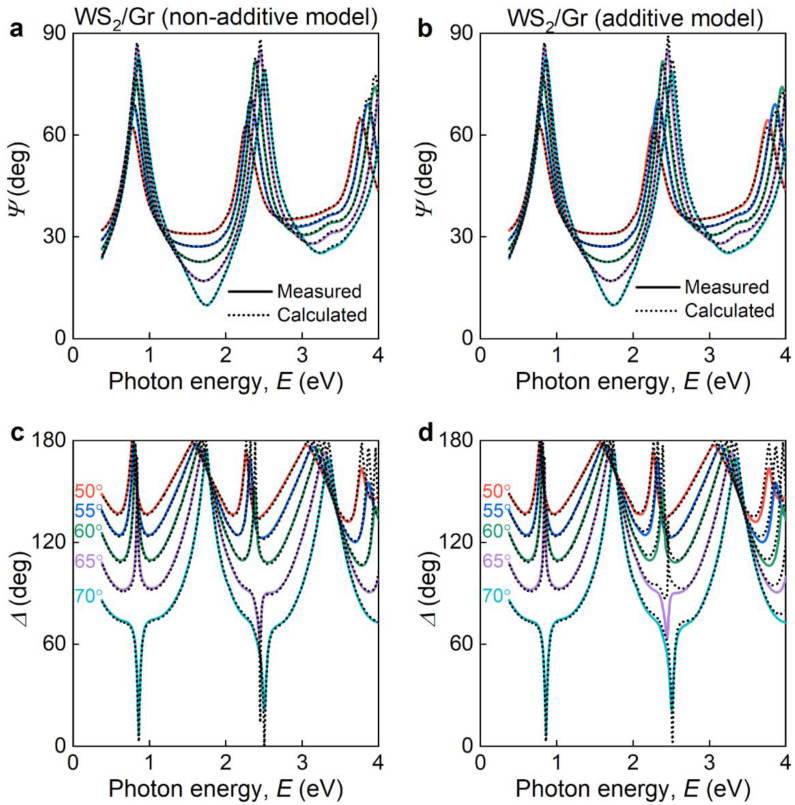
Ellipsometry spectra of WS_2_/Gr heterostructures. Comparison of non-additive and additive optical models for (**a**,**b**) *Ψ* and (**c**,**d**) Δ. Red, blue, green, violet, and cyan lines correspond to 50°, 55°, 60°, 65°, and 70° incident angles, respectively.

**Figure A4:**
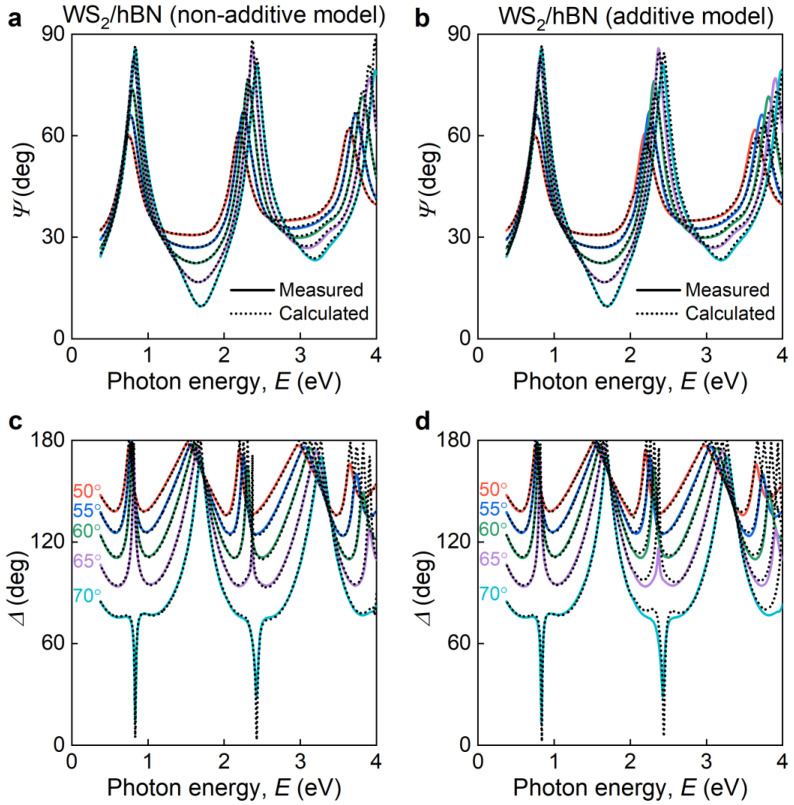
Ellipsometry spectra of WS_2_/hBN heterostructures. Comparison of non-additive and additive optical models for (**a**,**b**) *Ψ* and (**c**,**d**) Δ. Red, blue, green, violet, and cyan lines correspond to 50°, 55°, 60°, 65°, and 70° incident angles, respectively.

Similar to MoS_2_ heterostructures (see Section 2, we determined the optical constants of WS_2_ in the WS_2_/Gr and WS_2_/hBN heterostructures (Figure 4) from ellipsometry spectra (Figure A3 and Figure A4). However, in contrast to MoS_2_, the refractive index (Figure 4a) and extinction coefficient (Figure 4b) of WS_2_ demonstrate slight alterations, compared to pristine WS_2_. These observations are consistent with Raman findings (Figure 2b). Furthermore, if we take the original optical constants of WS_2_ to calculate ellipsometric parameters *Ψ* and Δ, then they will be close to the measured one, as seen from Figure A3 and Figure A4. Thus, although non-additive effects are present in WS_2_ heterostructures (Figure 4), the additive optical model retains high predictive capability. Hence, for computations of WS_2_ heterostructures in photonic devices, one can use both additive and non-additive optical models.

**Figure 5:**
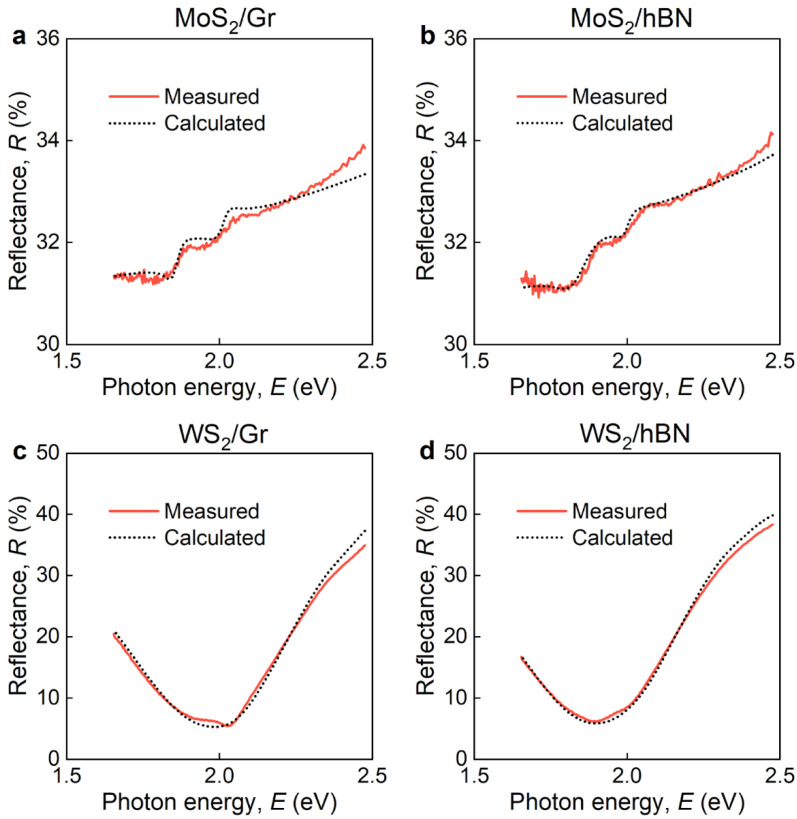
Measured and calculated reflectance spectra for (**a**) MoS_2_/Gr, (**b**) MoS_2_/hBN, (**c**) WS_2_/Gr, and (**d**) WS_2_/hBN heterostructures.

Apart from ellipsometry measurements, we also measured reflectance (Figure 5) of all heterostructures for unambiguous verification of found optical response, presented in Figure 3 and Figure 4. From Figure 5, we clearly see a good agreement between experimental and transfer matrix [63] calculated spectra. They additionally proved our conclusions about modified optical response of MoS_2_ and WS_2_ in heterostructures.

**Figure 6:**
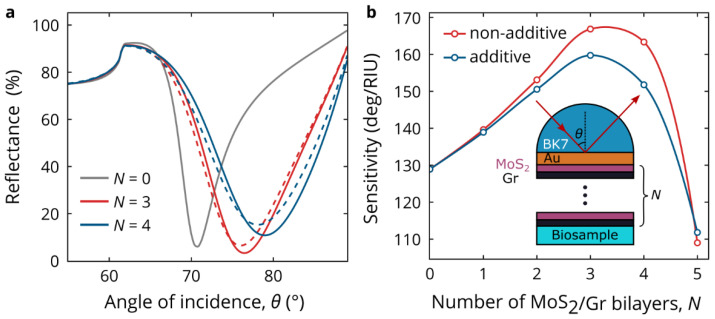
SPR biosensor performance. (**a**) Angle-resolved reflectance for different number *N* of MoS_2_/Gr bilayers deposited on gold layer. Dashed and solid lines were calculated using the additive and non-additive dielectric functions of MoS_2_, correspondingly. (**b**) Angular sensitivity of the biosensor as a function of *N* calculated with the additive (blue line) and non-additive (red line) dielectric functions of MoS_2_. Inset: schematic view of the studied biosensor. The thickness of gold is 40 nm, and the operating wavelength is 635 nm.

Finally, to illustrate the practical significance of observed modifications in the optical properties of TMDCs in the heterostructures, we theoretically studied a biosensor based on the surface plasmon resonance (SPR). To improve characteristics of the biosensor, we covered the surface of gold with a periodical MoS_2_/Gr heterostructure. Here, MoS_2_ is employed as a high-index material, whereas the topmost graphene sheet can serve as an effective linking layer [64,65]. We found that the optical sensitivity of the SPR biosensor is enhanced by deposition of the MoS_2_/Gr heterostructure, reaching as high as 167 deg/RIU with three added bilayers (Figure 6b). At the same time, the use of additive (unchanged) optical constants leads to a 4% lower optical sensitivity of 160 deg/RIU. In addition, the resonance dip position and magnitude depend on the optical properties of MoS_2_ (Figure 6a).

## 4. Conclusions

To summarize, we investigated the optical response of van der Waals (vdW) heterostructures on the example of MoS_2_/Gr, MoS_2_/hBN, WS_2_/Gr, and WS_2_/hBN. We observed a noticeable change in excitonic optical response in two-dimensional (2D) MoS_2_ and WS_2_, attributed to vdW interaction between layers in heterostructures. In detail, excitons in MoS_2_ demonstrate not only modification in oscillator strength, but also peak position shift and emergence of new excitonic peaks, resulting from a dark (light-inaccessible) to bright (light-accessible) exciton switch in the presence of graphene. On the other hand, excitons in WS_2_ show alterations only in oscillator strength and broadening, suggesting a different vdW interaction between Gr and hBN with MoS_2_ and WS_2_. Hence, our results indicate nontrivial influence of heterostructures on optical constants, which may be used to manipulate the optical response of vdW photonic [66,67,68] and optoelectronic [69,70,71] elements. Finally, it gives a positive answer to the question about non-additive effects of vdW heterostructures on the dielectric function of 2D materials. It offers an indispensable degree of freedom for photonic engineers, but complicates the computation of vdW heterostructures’ optical performance since each vdW configuration has a unique optical response, which does not decompose into individual monolayer responses.

## Data Availability

The data presented in this study are available upon reasonable request from the corresponding author.
